# Prevalence of impaired renal function among rural and urban populations: findings of a cross-sectional study in Malawi

**DOI:** 10.12688/wellcomeopenres.15255.1

**Published:** 2019-06-10

**Authors:** Wisdom P Nakanga, Josephine E Prynn, Louis Banda, Robert Kalyesubula, Laurie A Tomlinson, Moffat Nyirenda, Amelia C Crampin

**Affiliations:** 1 Malawi Epidemiology and Intervention Research Unit, Karonga, Malawi; 2 University of Exeter, Stocker Road, Exeter, EX4 4QD, UK; 3 London School of Hygiene & Tropical Medicine, Keppel Street, London, WC1E 7HT, UK; 4 MRC/UVRI & LSHTM Uganda Research Unit, Plot 51-59 Nakiwogo Road, Entebbe, Uganda

**Keywords:** Kidney Disease, Epidemiology, Sub Saharan Africa

## Abstract

**Background**: Sub-Saharan Africa faces region-specific risk factors for chronic kidney disease (CKD), such as nephrotoxic herbal medicines, antiretroviral therapy and infections, in addition to hypertension and diabetes. However, large epidemiological studies from this area are scarce.

**Methods**: In a cross-sectional survey of non-communicable diseases, we conducted a prevalence sub-study of CKD in two Malawian populations. Study participants (N=5264) of 18 years of age and above were recruited and data on demographics and CKD risk factors were collected. Glomerular filtration rate was estimated (eGFR) using the CKD-EPI equation.

**Results**: The prevalence of eGFR<60ml/min/1.73m ^2^ was 1.4% (95% CI 1.1 – 1.7%) and eGFR<90ml/min/1.73m ^2^ was 20.6% (95% CI 19.5 – 21.7%). The rural area had higher age-standardized prevalence of both eGFR<60ml/min/1.73m ^2^, at 1.8% (95% CI 1.4 – 2.3) and eGFR <90 ml/min/1.73m², at 21.1% (95% CI 19.9 – 22.3), than urban location, which had a prevalence of 1.5%, (95% CI 1.0 – 2.2) and 19.4% (95% CI 18.0 – 20.8), respectively, with overlapping confidence intervals. The prevalence of CKD was lower in females than in males in both rural and urban areas. Older age (p < 0.001), a higher level of education (p = 0.03) and hypertension (p < 0.001) were associated with a higher adjusted odds ratio (aOR) of low eGFR. Diabetes was associated with a reduced aOR of eGFR<90ml/min/1.73m ^2^ of 0.69 (95% CI 0.49–0.96; p=0.03). Of participants with eGFR<60ml/min/1.73m ^2^, 14 (19.4%) had no history of hypertension, diabetes or HIV, while 36 (50%) had a single risk factor of being hypertensive.

**Conclusion** **s**: Impaired renal function is prevalent, but lower than expected, in rural and urban Malawi. Further research is needed to increase understanding of CKD incidence, survival and validation of eGFR calculations in this population.

## Introduction

Since 1990, the number of deaths from chronic kidney disease (CKD) has risen by 82%, representing the third largest increase in deaths, behind HIV/AIDS and diabetes ^1, 2^. CKD, described as abnormalities of kidney structure or function present for longer than three months, is increasingly being recognized as an emerging public health problem globally and a key determinant of poor health outcomes of millions of people ^3– 5^. Sub-Saharan Africa (sSA) faces region-specific risk factors for CKD, such as nephrotoxic herbal medicines, antiretroviral therapy and infections, in addition to hypertension and diabetes ^6, 7^. The few epidemiological studies in sSA show a large variation of CKD prevalence, ranging from 1.5% to 33.5% ^8^.

However, current knowledge about the prevalence of CKD and renal disease in Malawi, as in most sSA countries, is scarce ^9^. Published data on CKD have been based on opportunistic sampling, involving people seeking medical care, or of urban populations, who have a different epidemiological profile from the rural majority ^6, 10^. It is thought that glomerulonephritis and CKD of unknown origin (CKDu), might account for a larger proportion of the total CKD burden in rural or disadvantaged populations, whilst hypertension and diabetes are more prevalent in urban areas ^3, 11^.

The absence of community-based representative data on CKD is concerning, as strategies aimed at managing CKD in Africa critically depend on reliable assessment of the burden of the problem and the establishment of affordable early detection programs ^12^. As such, we conducted a cross-sectional survey to estimate and compare the prevalence of CKD in rural and urban Malawi and analyzed the factors associated with this condition.

## Methods

### Study setting

**Figure 1.**
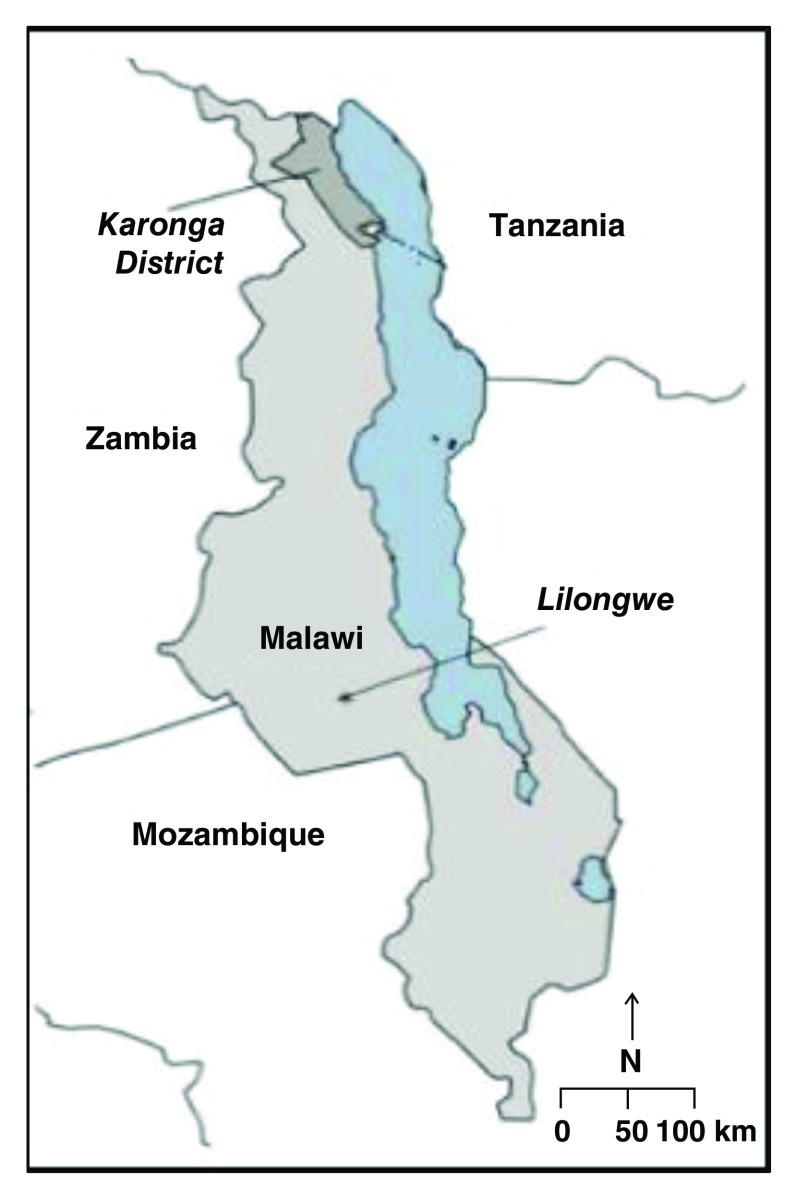
Map of Malawi Showing location rural Karonga and urban Lilongwe.

Participants were selected from a large cross-sectional population-based survey, designed to elucidate the burden and risk factors of non-communicable diseases in urban and rural Malawi ^13, 14^. The survey was conducted in a rural area with a population of 40,000 people within southern Karonga District and Area 25 in Lilongwe, the capital city ( Figure 1). Karonga is a lakeshore district, with a subsistence economy. Area 25 is an economically mixed urban economy with a population of around 66,000 individuals. Recruitment and data collection took place between May 16 ^th^ 2013 to February 8 ^th^ 2016.

### Recruitment and data collection

Detailed study procedures of the survey and characteristics of the two populations have been previously described ^13^ and all consenting adults age 18 years and over, residing within the defined study areas, were eligible to participate.

Briefly, the participants were visited in their homes. Written informed consent was obtained from all participants for interviewer-led questionnaires, documenting the participant’s socio-demographic information, lifestyle (e.g. smoking, alcohol, diet and physical activity), personal and family health history (e.g. hypertension, diabetes, heart disease and HIV) and current medications. Anthropometric measurements, measured twice by two study staff independently, and three seated blood pressure measurements, with 5 minute rests in between, were taken, adhering to standard protocols. A rapid HIV test was conducted, and results were given to the participants.

Blood was collected from an antecubital vein (following an overnight fast of at least 8h) in sodium fluoride tubes and plain serum tubes for fasting blood glucose (FBG) and serum creatinine tests, respectively. The samples were transported on the same day to the onsite project laboratory for the glucose assay and the serum was stored for creatinine analysis (mean time between collection and processing of 2.6h). We used the hexokinase glucose-6-phosphate dehydrogenase method using the AU480 Chemistry Analyzer (Beckman Coulter, California, USA), with sufficient sensitivity (range 0.555–44.4 mol/L) to determine glucose concentrations in the study samples.

Serum separation was done on a Sigma 6–16 centrifuge at 3000 RPM for 10 minutes. The separated serum was frozen in -80 freezer in 1.8ml Sarstedt tubes. On the day of analysis, the serum samples were taken out of the freezer, allowed to thaw at room temperature and then analysed on an AU480 analyser with ISE. Kinetic Jaffe (compensated) method, traceable to the isotope dilution mass spectrometry (IDMS) reference method was used. The AU480 analyser automatically computed the creatinine concentration of each sample and interference from protein was mathematically auto-corrected by the analyser by subtracting 18 μmol/L from each test result. The instrument was equipped with reagent OSR6178, System Calibrator Cat. No. 66300 traceable to the IDMS method via National Institute of Standards and Technology (NIST) Standard Reference Material (SRM) 967 and quality control material, all manufactured by Beckman Coulter (California, USA).

Participant ages were grouped into 10-year categories and education was categorized by the highest educational level achieved (no formal education, completed primary school standard 1–5, primary school standard 6–8, secondary, or tertiary). Occupation statuses were grouped into the following categories: not working, housework, farming or fishing, employed or self-employed. Household wealth scores were determined, based on the value of household assets, and then grouped into fifths across the total survey population. Physical activity was determined by self-reporting of the average duration (minutes) and intensity of exercise (pre-coded activities, grouped into high exertion, low exertion, or sedentary) in the previous week (both at work and during leisure time), used to establish whether participants met the World Health Organization (WHO) recommended level of activity per week. Alcohol consumption was divided into those who had consumed at least one alcoholic drink within the past 12 months and those who had not. Smoking status was divided into current smokers, ex-smokers and never-smokers.

Body mass index (BMI) categories were <18kg/m ^2^ (underweight), 18–24.99 kg/m ^2^ (healthy weight), 25–29.99 kg/m ^2^ (overweight) and >30 kg/m ^2^ (obese), in line with World Health Organization recommendations. Hypertension was defined as one or more of: systolic blood pressure of 140mmHg or more; diastolic blood pressure of 90 mmHg or more; and any use of antihypertensive medication in the past two weeks. Diabetes was defined as fasting plasma glucose of at least 7.0 mmol/L or any self-reported prior diagnosis of diabetes, regardless of whether the participant was taking anti-diabetic medication.

### Sampling participants for serum creatinine measurements

Serum samples were available for 24,780 participants; we sampled 5,264 serum samples for creatinine testing. With this sample size, we were able to determine a 0.1 standard difference, with power of 80% and p value of 0.01. Our sample was stratified by age (18–29, 30–39, 40–49, 50+), sex and site. We over-sampled older (aged 40 years plus) age groups in order to increase the numbers of individuals with impaired renal function to facilitate further analyses and prioritized participants recruited later in the study over those recruited earlier. Specifically, we tested 100 of each age/sex/site combination group for both 2015 and 2014, the remaining samples coming from participants aged 40 and older from 2015.

### Method of calculating of eGFR

We used the CKD-EPI equation to calculate eGFR:

eGFR = 141 × min(S _Cr_/K, 1) ^α^ × max(S _Cr_/K, 1) ^-1.209^ × 0.993 ^Age^ × 1.018[if female]

Where S _Cr_ is serum creatinine in µmol/L; K is 61.9 for females and 79.6 for males; α is -0.329 for females and -0.411 for males; Min indicates the minimum of S _Cr_/K or 1, and max indicates the maximum of S _Cr_/L or 1.

Using the this equation, not correcting for race, renal function was categorized as: eGFR <60 ml/min/1.73 m ^2^ (very low eGFR, representing CKD), <90 ml/min/1.73 m ^2^ (low GFR) and ≥90 ml/min/1.73 m ^2^ (normal), based on the National Kidney Foundation guidelines.

### Statistical analyses

We calculated the age-specific prevalence of eGFR<60ml/min/1.73m ^2^ and eGFR<90ml/min/1.73m ^2^ according to rural or urban residency and sex and estimated population prevalence, weighted to represent the age distribution of the underlying study population, using age groups of 18–29, 30–39, 40–49, and 50+, in line with the strategy used for sampling for the creatinine assay. In addition, the age-standardized prevalence (to the INDEPTH Standard population distribution 2000 – 2005 ^15^) was estimated.

**Figure 2.**
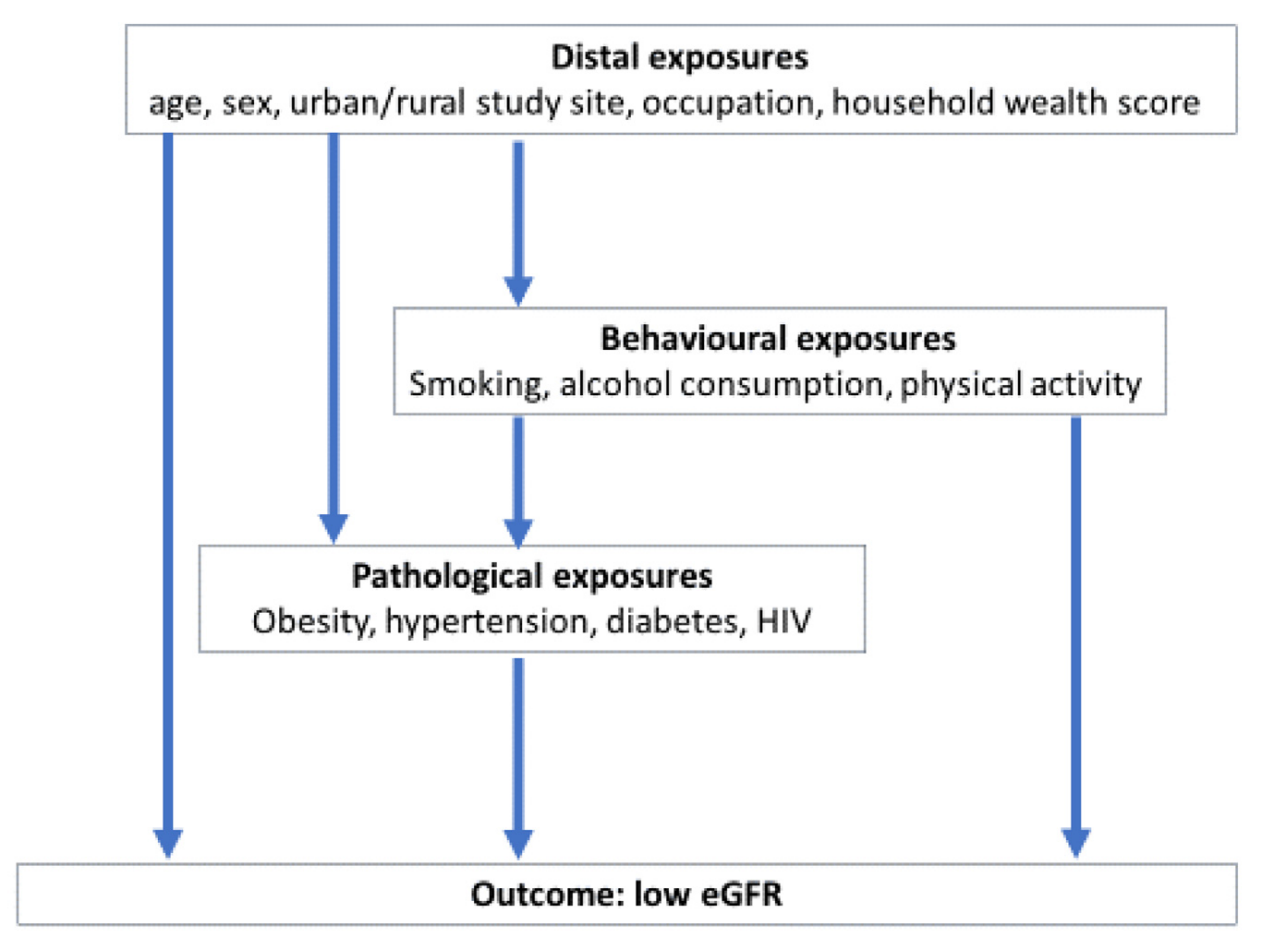
Conceptual framework of exposures potentially contributing to low estimated glomerular filtration rate (eGFR).

We conducted multivariable logistic regression to examine the association of different exposures with having an eGFR<90ml/min/1.73m ^2^. Exposures were considered in a conceptual framework of distal exposures (age, sex, urban or rural study site, education, occupation and household wealth score), behavioral exposures (smoking, alcohol consumption and physical activity) and pathological exposures (obesity, hypertension and impaired fasting glucose), displayed in Figure 2. We report the odds ratios (ORs) with 95% confidence intervals (CIs) for each of these exposures, adjusted for age and sex only, for distal exposures only, and for both distal and behavioral exposures. Complete case analysis was used as a strategy to deal with missing data. All analysis was done using Stata version 15.0 (StataCorp, College Station, TX).

### Ethical statement

All participants gave written consent to participate. Ethical approval was obtained from Malawi National Health Sciences Research Committee protocol number #1072, and London School of Hygiene and Tropical Medicine (UK) Ethics Committee protocol number #6303.

## Results

### Participant demographic characteristics

The creatinine assay was conducted on serum from 5264 participants. BMI was missing for 123 participants, mainly as it was not calculated in pregnant women. Blood pressure and diabetes data were missing for 3 and 2 participants, respectively. HIV status was not available for 1382 participants.

Socio-demographic characteristics of the participants are described in Table 1. 55.2% were from the rural area, 27.6% were between age 18 to 29, and 43.4% were farmers or fishermen. The rural sample was generally poorer, less well educated, and most participants were farmers or fishermen. The urban sample comprised more overweight or obese participants and had a higher prevalence of hypertension and diabetes.

**Table 1.** Baseline characteristics of the stratified sample by urban and rural study site.

	Rural		Urban	
	Female	Male	Female	Male
	n (%)	n (%)	n (%)	n (%)
**Age**				
18–29	388 (24.5)	318 (24.3)	447 (32.2)	302 (31.2)
30–39	327 (20.7)	304 (21.7)	329 (23.7)	222 (22.9)
40–49	367 (23.2)	316 (23.5)	328 (23.6)	222 (22.9)
50–59	255 (16.1)	190 (15.3)	177 (12.7)	111 (11.5)
60–69	152 (9.6)	92 (8.4)	74 (5.3)	80 (8.3)
70+	92 (5.8)	105 (6.8)	34 (2.5)	32 (3.3)
**Household possession score**				
1 (poorest)	419 (26.5)	319 (24.1)	126 (9.1)	99 (10.2)
2	450 (28.5)	380 (28.7)	121 (8.7)	118 (12.2)
3	297 (18.8)	258 (19.5)	253 (18.2)	175 (18.1)
4	332 (21.0)	291 (22.0)	416 (30.0)	234 (24.2)
5 (wealthiest)	83 (5.3)	77 (5.8)	473 (34.1)	343 (35.4)
**Occupation**				
Farming/fishing	1297 (82.0)	966 (72.9)	14 (1.0)	6 (0.6)
Self-employed	113 (7.2)	126 (9.6)	308 (22.2)	245 (25.3)
Employed	30 (1.9)	93 (7.0)	178 (12.8)	378 (39.0)
Not working	63 (4.0)	135 (10.2)	283 (20.4)	271 (28.0)
Housework	78 (4.9)	5 (0.4)	606 (43.6)	69 (7.1)
**Education**				
No formal	145 (9.2)	30 (2.3)	115 (8.3)	24 (2.5)
Standard 1–5	337 (21.3)	169 (12.8)	154 (11.1)	52 (5.4)
Standard 6–8	843 (53.3)	636 (48.0)	345 (24.8)	199 (20.5)
Secondary	243 (15.4)	459 (34.6)	602 (43.3)	474 (48.9)
Tertiary	13 (0.8)	31 (2.3)	173 (12.5)	220 (22.7)
**Alcohol consumption**				
No alcohol in past year	1509 (95.5)	787 (59.4)	1319 (95.0)	635 (65.5)
Alcohol in past year	72 (4.6)	538 (40.6)	70 (5.0)	334 (34.5)
**Smoking**				
Never smoked	1581 (100)	1071 (80.8)	1380 (99.4)	788 (81.3)
Ex-smoker	0 (0)	65 (4.9)	6 (0.4)	88 (9.1)
Current smoker	0 (0)	189 (14.3)	3 (0.2)	93 (9.6)
**Physical activity level**				
Did not meet WHO recommendations	26 (1.6)	36 (2.7)	31 (2.2)	50 (5.2)
Met WHO recommendations	1555 (98.4)	1289 (97.3)	1358 (97.8)	919 (94.8)
**Body Mass Index (kg/m ^2^) ^1^**				
<18	75 (5.0)	80 (6.1)	36 (2.7)	36 (3.7)
18–24.99	1029 (68.1)	1110 (83.9)	598 (44.7)	679 (70.1)
25–29.99	290 (19.2)	118 (8.9)	370 (27.7)	196 (20.2)
30+	118 (7.8)	15 (1.1)	333 (24.9)	58 (6.0)
**Hypertension**				
Hypertension	262 (16.6)	195 (14.7)	305 (22.0)	217 (22.4)
No hypertension	1317 (83.4)	1129 (85.3)	1084 (78.0)	752 (77.6)
**Diabetes**				
Diabetes	56 (3.5)	50 (3.8)	74 (5.3)	53 (5.5)
No diabetes	1525 (96.5)	1275 (96.2)	1314 (94.6)	915 (94.4)
**HIV status**				
HIV positive	140 (14.3)	101 (13.7)	140 (10.7)	72 (8.4)
HIV negative	840 (85.7)	638 (86.3)	1162 (89.3)	789 (91.6)

^1^Missing data on 123 participants (69 rural women, 2 rural men, 52 urban women). ^2^Missing data on 3 participants (2 rural women, 1 rural man). ^3^Missing data on 2 participants (1 urban woman, 1 urban man). ^4^Missing data on 1382 participants (601 rural women, 586 rural men, 87 urban women, 108 urban men).

### Crude and age-specific prevalence

**Figure 3.**
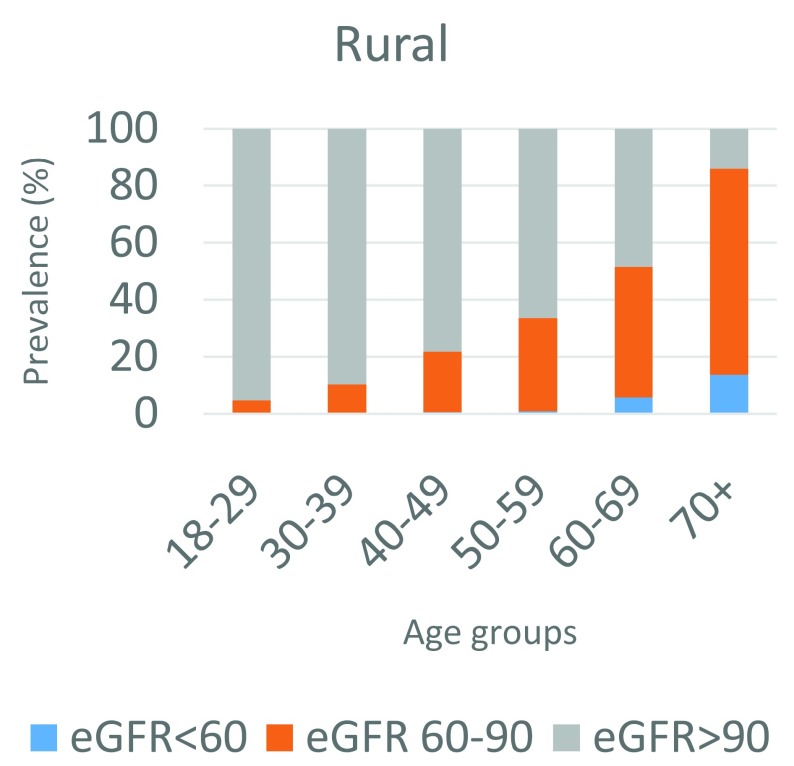
Age specific prevalence of low estimated glomerular filtration rate (eGFR) in the rural site.

**Figure 4.**
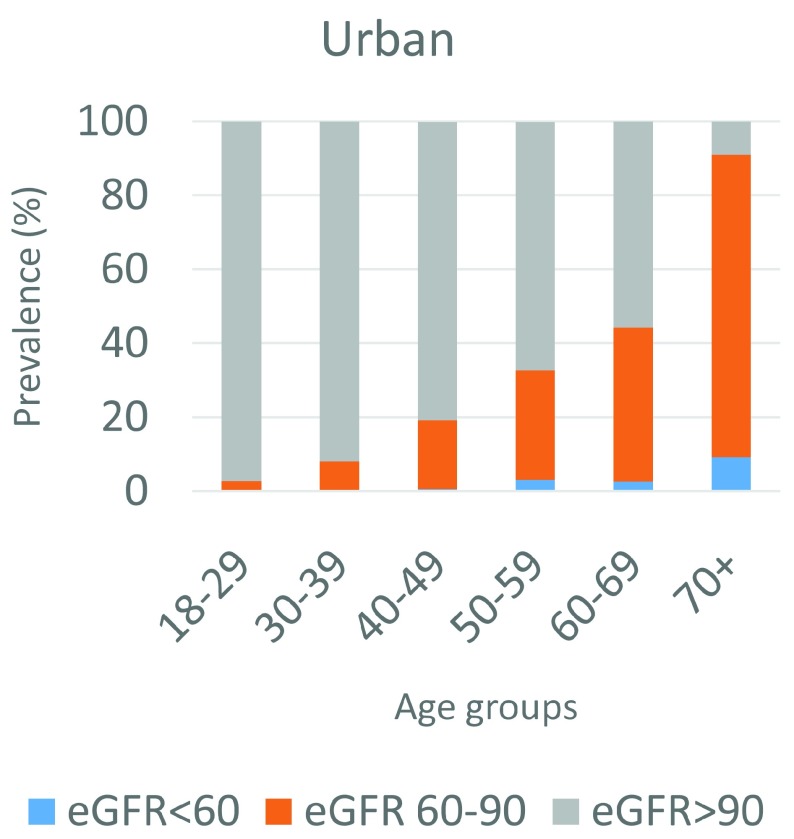
Age-specific prevalence of low estimated glomerular filtration rate (eGFR) in the urban study site.

The prevalence of eGFR<60ml/min/1.73m ^2^ in our sample overall was 1.4% (95% CI 1.1 – 1.7) and the prevalence of eGFR<90ml/min/1.73m ^2^ was 20.6% (95% CI 19.5 – 21.7). This is shown by age and urban or rural location in Table 2 and Table 3 and Figure 3 and Figure 4. The rural area had higher prevalence of both eGFR<60ml/min/1.73m ^2^, at 1.8% (95% CI 1.4 – 2.3), and eGFR<90ml/min/1.73m ^2^, at 21.1% (95% CI 19.9 – 22.3), than the urban location, which had a prevalence of 1.5% (95% CI 1.0 – 2.2) and 19.4% (95% CI 18.0 – 20.8), respectively, although with overlapping confidence intervals when standardized to the INDEPTH standard age distribution. The prevalence of eGFR<60ml/min/1.73m ^2^ was also slightly lower in females than in males in both rural and urban areas.

**Table 2.** Prevalence (%) of chronic kidney disease (CKD) (eGFR<60 ml/min/1.73m ^2^).

Prevalence	Rural			Urban		
	Female	Male	Total	Female	Male	Total
	27/1581	23/ 1325	50/ 2906	10/ 1389	12/ 969	22/ 2358
Stratified study population	1.7 (1.2-2.5)	1.7 (1.2-2.6)	1.7 (1.3-2.3)	0.7 (0.4-1.3)	1.2 (0.7-2.2)	0.9 (0.6-1.4)
Standardized to age distribution of underlying population	1.3 (0.9-1.9)	1.3 (0.9-1.9)	1.4 (1.0-1.8)	0.4 (0.2-0.7)	0.7 (0.4-1.2)	0.6 (0.4-0.8)
Standardized to INDEPTH standard age distribution	1.5 (1.1-2.1)	1.8 (1.2-2.6)	1.8 (1.4-2.3)	1.0 (0.5-0.2)	1.6 (1.0-2.7)	1.5 (1.0-2.2)

**Table 3.** Prevalence (%) of low estimated glomerular filtration rate (eGFR<90 ml/min/1.73m ^2^).

Prevalence	Rural			Urban		
	Female	Male	Total	Female	Male	Total
	399/ 1581	291/ 1325	690/ 2906	213/ 1389	180/ 969	393/ 1965
Stratified study population	25.24 (23.2-27.4)	21.96 (19.8-24.3)	23.7 (22.2-25.3)	15.3 (13.5-17.3)	18.6 (16.3-21.2)	16.7 (15.2-18.2)
Standardized to age distribution of underlying population	19.9 (18.4-21.5)	18.0 (16.4-19.7)	19.1 (18.0-20.3)	10.6 (9.4-12.0)	12.4 (10.7-14.3)	11.3 (10.3-12.5)
Standardized to INDEPTH standard age distribution	22.2 (20.1-23.9)	19.5 (17.8-21.4)	21.1 (19.9-22.3)	18.7 (16.9-21.6)	20.2 (18.1-22.3)	19.4 (18.0-20.8)

### Exposures associated with low eGFR

Low eGFR was strongly associated with increasing age ( Table 4), with participants in the over-70 age group having 148 times the odds of low eGFR than those in the 18–39 age group, when adjusted for distal and behavioral exposures (95% CI 90.88 – 242.27). Other exposures associated with low eGFR in the fully-adjusted model were having a higher education level (p=0.03), being a farmer or fisherman, and hypertension (p<0.001). Being underweight was associated with reduced odds of low eGFR (p=0.03). In our sample, diabetes was associated with a reduced adjusted odds ratio (aOR) of eGFR<90ml/min/1.73m ^2^, with an aOR of 0.57 (95% CI 0.40 – 0.81; p=0.001), adjusting for distal and behavioral exposures. Place of residence was associated with eGFR<90ml/min/1.73m ^2^ with an age- and sex-adjusted OR of 0.84 (95% CI 0.72 – 0.98; p=0.03) for urban residence, with rural as the reference. The association was no longer seen when also adjusting for distal or behavioral exposures.

**Table 4.** Factors associated with estimated glomerular filtration rate (eGFR) <90 adjusted for age and sex only, adjusted for distal factors, and adjusted for both distal and behavioral factors.

	Adjusted for age and sex only		Adjusted for distal factors (age, sex, site, education, occupation, wealth score)		Adjusted for distal and behavioral factors (age, sex, site, occupation, wealth score, smoking, alcohol and physical activity), and diabetes and hypertension	
	OR (95% CI)	p ^1^	OR (95% CI)	p ^1^	OR (95% CI)	p ^1^
**Sex**						
Female	1	0.26	1	0.02	1	0.39
Male	0.91 (0.78-1.07)		0.81 (0.68-0.96)		0.92 (0.76-1.12)	
**Age group**						
18–29	1	<0.001	1.00	<0.001	1.00	<0.001
30–39	2.61 (1.87-3.66)		2.59 (1.83-3.66)		2.50 (1.77-3.54)	
40–49	6.77 (4.99-9.19)		6.68 (4.86-9.18)		6.27 (4.55-8.64)	
50–59	12.86 (9.41-17.57)		12.98 (9.37-18.00)		11.32 (8.09-15.83)	
60–69	24.71 (17.67-34.56)		26.01 (18.37-36.83)		20.69 (14.35-29.83)	
70+	176.55 (112.37-277.37)		199.27 (124.15-319.84)		148.38 (90.88-242.27)	
**Site**						
Rural	1	0.03	1	0.92	1	0.82
Urban	0.84 (0.72-0.98)		1.01 (0.77-1.34)		0.97 (0.73-1.28)	
**Household wealth score**						
1 (poorest)	1	0.53 ^*^	1.00	0.54 ^*^	1.00	0.78 ^*^
2	1.12 (0.87-1.44)		1.12 (0.87-1.44)		1.10 (0.85-1.42)	
3	1 .00 (0.77-1.29)		1.03 (0.79-1.35)		1.00 (0.76-1.31)	
4	1.06 (0.83-1.35)		1.06 (0.83-1.37)		1.02 (0.79-1.32)	
5 (wealthiest)	1.14 (0.88-1.48)		1.15 (0.86-1.54)		1.11 (0.82-1.49)	
**Education**						
No formal	0.82 (0.60-1.11)	0.03 ^*^	0.86 (0.63-1.19)	0.03 ^*^	0.85 (0.62-1.17)	0.03 ^*^
Standard 1–5	0.88 (0.69-1.11)		0.87 (0.69-1.11)		0.90 (0.71-1.14)	
Standard 6–8	1.00		1.00		1.00	
Secondary	0.92 (0.75-1.12)		1.01 (0.81-1.24)		1.01 (0.82-1.26)	
Tertiary	1.42 (1.07-1.90)		1.47 (1.04-2.07)		1.53 (1.08-2.17)	
**Occupation**						
Not working	1.25 (0.89-1.76)	<0.001	1.17 (0.83-1.66)	0.003	1.09 (0.77-1.56)	0.003
Housework	0.86 (0.63-1.18)		0.89 (0.65-1.23)		0.87 (0.63-1.20)	
Farming/fishing	1.44 (1.13-1.84)		1.55 (1.14-2.12)		1.58 (1.15-2.15)	
Self-employed	1.00		1.00		1.00	
Employed	1.58 (1.18-2.13)		1.42 (1.04-1.93)		1.37 (1.00-1.88)	
**Smoking status**						
Never smoker	1	0.02	1	0.03	1	0.17
Ex-smoker or current smoker	0.70 (0.52-0.94)		0.71 (0.53-0.97)		0.80 (0.59-1.10)	
**Alcohol consumption**						
Never	1	0.06	1	0.05	1	0.11
Rarely/often	0.81 (0.65-1.01)		0.80 (0.64-1.00)		0.83 (0.65-1.04)	
**Physical activity ^2^**						
Did not meet WHO recommendation	1.48 (0.96-2.28)	0.08	1.44 (0.93-2.23)	0.10	1.32 (0.85-2.07)	0.22
Met WHO recommendation	1.00		1.00		1.00	
**BMI (kg/m ^2^) ^3^**						
<18	0.58 (0.38-0.89)	0.03	0.57 (0.38-0.87)	0.009	0.62 (0.41-0.94)	0.04
18–24.99	1		1.00		1.00	
25–29.99	1.12 (0.92-1.36)		1.19 (0.97-1.45)		1.16 (0.94-1.43)	
30+	1.04 (0.82-1.34)		1.13 (0.87-1.48)		1.04 (0.79-1.37)	
**Hypertension ^4^**						
No hypertension	1	<0.001	1	<0.001	1	<0.001
Hypertension	1.57 (1.30-1.88)		1.69 (1.40-2.05)		1.77 (1.46-2.16)	
**Diabetes ^5^**						
No diabetes	1	0.03	1	0.03	1	0.001
Diabetes	0.71 (0.51-0.98)		0.70 (0.50-0.97)		0.57 (0.40-0.81)	
**HIV**						
Negative	1	0.50	1	0.52	1	0.29
Positive	1.09 (0.84-1.41)		1.09 (0.84-1.42)		1.16 (0.89-1.51)	

1. Likelihood ratio tests for association. Values marked with * are test-for-trend p-values. 2. High defined as meeting recommended WHO physical activity levels. 3. Missing data on 123 participants 4. Defined as blood pressure ≥140/90 or on antihypertensive medication, missing data on 3 participants 5. Defined as fasting plasma glucose≥7.1 or self-report, missing data on 3 participants BMI, body mass index; OR, odds ratio; CI, confidence intervals

Of those who had eGFR<60ml/min/1.73m ^2^, 14 (19.4%) had no history of hypertension, diabetes or HIV ( Table 5). Thirty-six (50.0%) of the participants with eGFR<60ml/min/1.73m ^2^ had a single risk factor of being hypertensive, eight (11.1%) had a single risk factor of HIV and nine (19.4%) had two known risk factors of CKD.

**Table 5.** Number and proportion of participants with known hypertension, diabetes and HIV with low and very low estimated glomerular filtration rate (eGFR) ^1^.

		eGFR>90	eGFR 60-90	eGFR<60	Total
		n (%)	n (%)	n (%)	n (%)
**No known risk factors**	No hypertension, diabetes, or HIV	3259 (88.0%%)	547 (54.1%)	14 (19.4%)	3820 (72.6%)
**Single known risk factor**	Hypertension only	433 (10.4%)	315 (31.2%)	36 (50.0%)	784 (14.9%)
	Diabetes only	58 (1.4%)	14 (1.4%)	0	72 (1.4%)
	Known HIV only	304 (7.3%)	68 (6.7%)	8 (11.1%)	380 (7.2%)
**2 known risk factors**	Hypertension and diabetes	83 (2.0%)	43 (4.3%)	9 (12.5%)	135 (2.6%)
	Hypertension and HIV	25 (0.6%)	17 (1.7%)	5 (6.9%)	47 (0.9%)
	Diabetes and HIV	11 (0.3%)	2 (0.2%)	0	13 (0.2%)
**3 known risk factors**	Hypertension, diabetes and HIV	8 (0.2%)	5 (0.5%)	0	13 (0.2%)
	Overall	4181	1011	72	5264

1 Participants with missing data on any of the three risk factors examined were classified as not having known risk factor

## Discussion

In this large, population-based, cross-sectional stratified sample, we found an overall standardized prevalence of eGFR<90ml/min/1.73m ^2^ of 20.6% and eGFR<60ml/min/1.73m ^2^ of 1.4% using the CKD-EPI equation. A study conducted in Lilongwe by Glaser *et al.* found a prevalence of eGFR<60ml/min/1.73m ^2^ of 3.1%, in individuals seeking HIV testing in urban Malawi ^8^. These estimates are comparable to those found in other regional studies and validate a growing body of research indicating presence of undiagnosed CKD at population level ^16, 17^.

In our study, low eGFR was associated with occupation (farmers/fisherman more likely to have decreased eGFR), increasing age, and hypertension and was inversely associated with being underweight in fully adjusted analyses. These associations are similar to those found by Kalyesubula *et al*. in rural Uganda ^17^. The high prevalence of CKD amongst rural participants and farmers has been documented in other literature ^18^. Rural agricultural communities from Central America, Egypt, India, and Sri Lanka have reported a high prevalence of CKDu of unknown etiology, and a clinical presentation suggestive of interstitial nephritis, confirmed by renal biopsies, with male farm workers affected disproportionately ^3^. The rural site investigated in the study has a high population of males working in agricultural fields in similar circumstances to those studied in other regions and we could postulate that it has similar epidemiology, but more research is required. Alternately, the result could be confounded by higher creatinine levels due to the high muscle mass in this physically active group. Similarly, the lower odds of CKD in underweight participants may reflect reduced muscle bulk with lower creatinine production. As expected, loss of renal function in our study was associated with an older age. CKD may be a consequence of expected age-related renal function decline, accelerated in hypertension, diabetes, obesity, and primary renal disorders ^5^. Similar studies in other settings have shown that an age of over 60, female gender, low educational status, increased waist circumference, hypertension, and diabetes were associated with low eGFR ^16^. The high prevalence of CKD amongst hypertensive participants is in keeping with several similar studies carried out in sSA ^1, 6^. Inadequate blood pressure control may explain the prevalence of CKD observed ^7^; however, poor renal function may also lead to an increase in blood pressure.

Our study also suggests an inverse association between renal impairment and diabetes. A survivor bias in the cross-sectional design may explain this association ^16^, as it is possible that people with diabetes die before developing renal impairment, or that people with diabetes who develop renal impairment have very poor survival. Additionally, hyperfiltration is a feature of early diabetic nephropathy, associated with a high eGFR. Early diabetic kidney disease would not be picked up from the eGFR alone, and may bias the results in the direction of diabetes being protective for kidney disease ^19^.

The results of the study are useful because of the large sample size, the study of the general population rather than patients in a clinical setting, and the ability to compare between rural and urban populations. The previous study conducted in Lilongwe recruited participants attending a HIV Testing and Counselling Centre and may not be a good estimate of population prevalence. In particular, the prevalence of HIV in the study was greater than in the general population and the HIV-negative patients may have been symptomatic for other conditions, prompting self-referral for HIV testing ^9^. As our study population is of a similar age and sex structure to the Malawian population and represent typical rural and urban populations, our results are likely to be representative of the rest of the country, and possibly to other similar populations in low resource and low income countries ^1^.

A limitation of our study was relying on serum creatinine for the measurement of CKD with no urine albumin:creatinine ratio, since classification of CKD stages one and two includes other evidence of renal damage including proteinuria, haematuria, or evidence of abnormal anatomy or systemic disease. Furthermore, CKD can only be formally diagnosed if low eGFR and/or a high urine albumin:creatinine ratio have been found at two different times, with at least a three month gap ^2^, and 30–40% of patients labelled as CKD stage 3A when the diagnosis is based on only a single eGFR determination move to ‘normal’ ^2^. Therefore, the reported prevalence of CKD might be overestimated ^7^. However, as nearly all other prevalence studies have defined CKD based on a one-off measurement of eGFR <60 mL/min/1.73m ^2^, this does allow our results to be directly comparable to much of the published data ^2, 20^. Furthermore, the study used CKD-EPI equation in the determination of eGFR, which is considered to be a more accurate predictor of clinical risk than other serum-based equations, including the Modification of Diet in Renal Disease (MDRD) equation ^5^. However, the CKD-EPI equation has not been validated for the African population, so it is possible that calculated eGFR is not reflecting the underlying kidney function accurately in our population. Finally, cross-sectional studies do not allow one to derive a causal inference, which is particularly important when considering the relationship between hypertension, diabetes and CKD.

## Conclusion

Our study is the first to estimate population-level prevalence of CKD in urban and rural Malawi, and highlights that there is already a substantial burden of disease. With an ageing population and increasing prevalence of hypertension, this is likely to increase over the coming decades. Furthermore, in the absence of screening of high-risk individuals, diagnosis, and appropriate service provision, poor survival with CKD is likely and this estimated prevalence may mask a much higher incidence. Longitudinal research is urgently needed to increase understanding of CKD incidence, survival and validation of eGFR calculations in this population.

## Data availability

### Underlying data

The cross-sectional NCD data underlying this analysis are available for download via the LSHTM Data Compass Repository ( https://doi.org/10.17037/DATA.00000961). However, owing to data protection concerns, there are restrictions on access to this underlying data. Given their linkages to the longitudinal data produced by MEIRU, these data require anonymization using statistical disclosure control techniques to make them sufficiently safe for direct access. The anonymization process is scheduled for the last quarter of 2019. In the meantime, data access to bona fide researchers for specific research purposes is possible through completing a data request form on the LSHTM data compass repository. Once the anonymization process has been completed, direct access will be granted via the same repository.

## References

[1] Ephraim RK Biekpe S Sakyi SA: Prevalence of chronic kidney disease among the high risk population in South-Western Ghana; a cross sectional study. *Can J Kidney Health Dis.* 2015;2(1):40. 10.1186/s40697-015-0076-3 26535132 PMC4630826

[2] Jayasekara KB Dissanayake DM Sivakanesan R: Epidemiology of chronic kidney disease, with special emphasis on chronic kidney disease of uncertain etiology, in the north central region of Sri Lanka. *J Epidemiol.* 2015;25(4):275–80. 10.2188/jea.JE20140074 25787679 PMC4375281

[3] Garcia-Garcia G Jha V Tao Li PK: Chronic kidney disease (CKD) in disadvantaged populations. *Clin Kidney J.* 2015;8(1):3–6. 10.1093/ckj/sfu124 25713703 PMC4310427

[4] Adeniyi AB Laurence CE Volmink JA: Prevalence of chronic kidney disease and association with cardiovascular risk factors among teachers in Cape Town, South Africa. *Clin Kidney J.* 2017;10(3):363–369. 10.1093/ckj/sfw138 28621342 PMC5466082

[5] Hill NR Fatoba ST Oke JL: Global Prevalence of Chronic Kidney Disease - A Systematic Review and Meta-Analysis. *PLoS One.* 2016;11(7):e0158765. 10.1371/journal.pone.0158765 27383068 PMC4934905

[6] Kaze FF Meto DT Halle MP: Prevalence and determinants of chronic kidney disease in rural and urban Cameroonians: a cross-sectional study. *BMC Nephrol.* 2015;16(1):117. 10.1186/s12882-015-0111-8 26220538 PMC4518633

[7] Sumaili EK Cohen EP Zinga CV: High prevalence of undiagnosed chronic kidney disease among at-risk population in Kinshasa, the Democratic Republic of Congo. *BMC Nephrol.* 2009;10:18. 10.1186/1471-2369-10-18 19622160 PMC2724413

[8] Glaser N Deckert A Phiri S: Comparison of Various Equations for Estimating GFR in Malawi: How to Determine Renal Function in Resource Limited Settings? *PLoS One.* 2015;10(6):e0130453. 10.1371/journal.pone.0130453 26083345 PMC4470826

[9] Glaser N Phiri S Bruckner T: The prevalence of renal impairment in individuals seeking HIV testing in Urban Malawi. *BMC Nephrol.* 2016;17(1):186. 10.1186/s12882-016-0403-7 27875991 PMC5118906

[10] Singh M Magula NP Hariparshad S: A comparison of urban and rural patients with chronic kidney disease referred to Inkosi Albert Luthuli Central Hospital in Durban, South Africa. *African Journal of Nephrology.* 2017;20(1):34–38. 10.21804/20-1-1358

[11] Rajapakse S Shivanthan MC Selvarajah M: Chronic kidney disease of unknown etiology in Sri Lanka. *Int J Occup Environ Health.* 2016;22(3):259–264. 10.1080/10773525.2016.1203097 27399161 PMC5102238

[12] Abd ElHafeez S Bolignano D D'Arrigo G: Prevalence and burden of chronic kidney disease among the general population and high-risk groups in Africa: a systematic review. *BMJ Open.* 2018;8(1):e015069. 10.1136/bmjopen-2016-015069 29326180 PMC5780690

[13] Crampin AC Kayuni N Amberbir A: Hypertension and diabetes in Africa: design and implementation of a large population-based study of burden and risk factors in rural and urban Malawi. *Emerg Themes Epidemiol.* 2016;13:3. 10.1186/s12982-015-0039-2 26839575 PMC4736489

[14] Price AJ Crampin AC Amberbir A: Prevalence of obesity, hypertension, and diabetes, and cascade of care in sub-Saharan Africa: a cross-sectional, population-based study in rural and urban Malawi. *Lancet Diabetes Endocrinol.* 2018;6(3):208–222. 10.1016/S2213-8587(17)30432-1 29371076 PMC5835666

[15] Sankoh O Sharrow D Herbst K: The INDEPTH standard population for low- and middle-income countries, 2013. *Glob Health Action.* 2014;7:23286. 10.3402/gha.v7.23286 24679543 PMC3969509

[16] Singh NP Ingle GK Saini VK: Prevalence of low glomerular filtration rate, proteinuria and associated risk factors in North India using Cockcroft-Gault and Modification of Diet in Renal Disease equation: an observational, cross-sectional study. *BMC Nephrol.* 2009;10:4. 10.1186/1471-2369-10-4 19220921 PMC2663556

[17] Kalyesubula R Hau JP Asiki G: Impaired renal function in a rural Ugandan population cohort [version 2; peer review: 1 approved, 1 approved with reservations]. *Wellcome Open Res.* 2019;3:149 10.12688/wellcomeopenres.14863.2 31223661 PMC6560494

[18] Lebov JF Valladares E Peña R: A population-based study of prevalence and risk factors of chronic kidney disease in León, Nicaragua. *Can J Kidney Health Dis.* 2015;2:6. 10.1186/s40697-015-0041-1 25926994 PMC4414463

[19] Trevisan R Dodesini AR: The Hyperfiltering Kidney in Diabetes. *Nephron.* 2017;136(4):277–280. 10.1159/000448183 27978521

[20] Delanaye P Glassock RJ De Broe ME: Epidemiology of chronic kidney disease: think (at least) twice! *Clin Kidney J.* 2017;10(3):370–374. 10.1093/ckj/sfw154 28617483 PMC5466090

