# Supplementary material for: Prevalence of impaired renal function among rural and urban populations: findings of a cross-sectional study in Malawi
Source: Wellcome Open Res. 2019 Jun 10;4:92. [Version 1] doi: 10.12688/wellcomeopenres.15255.1 (PMC6798316; doi:10.12688/wellcomeopenres.15255.1)
Supplement: Peer review history

# Peer review: Reviewer response for version 1

**DOI:** 10.21956/wellcomeopenres.16651.r36520

**Published:** 2019-10-17

**Authors:** Nombulelo P Magula

**Affiliations:** 1 Department of Internal Medicine, Nelson R. Mandela School of Medicine, University of KwaZulu-Natal (UKZN), Durban, South Africa

The study compares renal function in a rural and urban population in sub-Saharan Africa. The authors have presented population data on renal function and risk factors.

 The work adds to an important body of knowledge. The rationale for the study is clearly articulated. The methodology is sound and the results very well presented and interpreted. 

 Some of the variables used need justification.

 The rationale to use the categorization of at least one alcoholic drink in the past 12 months versus no alcohol needs to be explained. The biological or clinical effects of one alcoholic drink in 12 months compared to an excessive alcohol drinker would be vastly different.

 In this paper, hypertension was defined as “…one or more of: systolic blood pressure of 140mmHg or more; diastolic blood pressure of 90 mmHg or more; and any use of antihypertensive medication in the past two weeks.” Yet in the published methods paper (Crampin AC et. al 2016 ^1^), this was defined as “three blood pressure measurements, with 5 min resting time in between,..., and the average of the last two readings was used”. The definition used in the methods of the two papers needs to be aligned. 

 The relationship found between diabetes and renal function is surprising and the attempt to explain the finding of diabetes being protective is reasonably explained. The limitation of urine examination not being done has been explicitly described.

 In diabetes, retinal changes occur before the onset of nephropathy and this clinical detail should be alluded to in the attempts to explain the unexpected  protective findings of diabetes. The lack of retinal examination in the study should be highlighted as a limitation in making the link between diabetes and nephropathy as found in this study. If the examination confirmed absence of retinopathy, then this would support a possibility that those with diabetes had early disease.

 The effects of HIV infection are alluded to in the background, however, the stage of HIV disease or effects of antiretroviral treatment has not been explored. This needs to be addressed in the limitations.

 Excellent work overall.

I confirm that I have read this submission and believe that I have an appropriate level of expertise to confirm that it is of an acceptable scientific standard.

## References

[1] : Hypertension and diabetes in Africa: design and implementation of a large population-based study of burden and risk factors in rural and urban Malawi. *Emerg Themes Epidemiol*.2016;13: 10.1186/s12982-015-0039-2 3 10.1186/s12982-015-0039-2 26839575PMC4736489

# Peer review: Reviewer response for version 1

**DOI:** 10.21956/wellcomeopenres.16651.r35849

**Published:** 2019-08-29

**Authors:** Richard K.D. Ephraim

**Affiliations:** 1 Department of Medical Laboratory Technology, University of Cape Coast, Cape Coast, Ghana

A very good work which adequately reviewed existing data and used a rigorous methodology to establish the prevalence of CKD in urban and rural malawi

 However, though the authors examined the population for the presence of hypertension and diabetes mellitus I did not see any data on the duration of the diabetes and hypertension and even the therapy these patients or participants used. I think this is relevant since earlier studies have established a relationship between duration of these chronic conditions and CKD as well as the medications and CKD

 Secondly the study of Ephraim *et al* ^1^ and also Janmohammed *et al* ^2^ **estimated or identified evidence of kidney damage by measuring microalbuminuria or dipstick proteinuria 

 I realized that the authors did not mention anything on evidence of renal damage. Authors should provide reasons for this omission.

 CKD diagnosis is either based on the KDIGO or KDOQI guidelines 

 Which of these did the authors use and why?

I confirm that I have read this submission and believe that I have an appropriate level of expertise to confirm that it is of an acceptable scientific standard, however I have significant reservations, as outlined above.

## References

[1] : Prevalence of chronic kidney disease among the high risk population in South-Western Ghana; a cross sectional study. *Can J Kidney Health Dis*.2015;2: 10.1186/s40697-015-0076-3 40 10.1186/s40697-015-0076-3 26535132PMC4630826

[2] : Prevalence of chronic kidney disease in diabetic adult out-patients in Tanzania. *BMC Nephrol*.2013;14: 10.1186/1471-2369-14-183 183 10.1186/1471-2369-14-183 24228774PMC3765892

# Peer review: Reviewer response for version 1

**DOI:** 10.21956/wellcomeopenres.16651.r35749

**Published:** 2019-08-15

**Authors:** Robert N. Peck

**Affiliations:** 1 Center for Global Health, Department of Medicine, Weill Cornell Medicine, New York City, NY, USA

In this large, population based, cross-sectional study of 5264 adults in urban and rural Malawi, the authors investigate the epidemiology of impaired renal function. The study population is a nested cohort within a much larger population based survey in Malawi that was conducted between 2013-2016. The outcome (impaired renal function) is defined by eGFR < 60 or <90 based on a single creatinine measurement. The methods are generally rigorous and well described and limitations are noted. The resulting analysis provides a useful snapshot of the distribution of kidney disease that will be valuable in guiding public health efforts to prevent and treat this important disease.

 I recommend that several major and minor revisions should be made to this manuscript before it is indexed. I have described these concerns below.

 Methods:

- Were the study areas truly either urban or rural? Were any of the areas peri-urban? is there any mining conducted in any of the study sites?
- A brief description of the sampling procedure that was used for selecting the 24,780 study participants from the source population should be provided in this manuscript. Authors can refer to the prior publications for details related to sampling procedure.
- What was the method for selecting the 5,264 serum samples that were tested from the 24,780 total study participants? The stratification is well described but was the sampling random within these strata? 
- 3 blood pressure measurements were taken. How many of these were used to calculate the average blood pressure? Did participants with elevated blood pressure have repeat measurements conducted according to the guidelines?
- Diagnosis of diabetes mellitus depended on fasting glucose. What methods were used to ensure that the participant had actually fasted?
- Why was the CKD EPI equation without race correction chosen for this analysis? References should be provided to support this choice since this is the primary outcome and multiple GFR calculator options exist. 

Statistical analysis/Interpretation

- Complete case analysis was used to deal with missing data. The pattern of missingness should be described in the methods section. Did missingness impact the selection of serum samples?
- 123 participants included in this analysis were pregnant. BMI was not calculated for these participants. Pregnancy is known to be related to eGFR (the outcome variable) and several of the exposure variables such as blood pressure. Could this be a hidden confounder in your analysis? What was the association between pregnancy and eGFR? 
- In Table 2, some of the superscripts listed in the footnote are not clearly visible within the table.

Conclusions

- If I am interpreting Table 5 correctly, 58/72 of the participants with eGFR < 60 had HTN. This point should be emphasized in the discussion and the implications for linking HTN and kidney disease screening should be discussed.
- Are the fisherman/farmers exposed to schistosomiasis? Could this or some other helminthic infection explain the link between fisherman/farmers and CKD? This possibility should be discussed and references should be listed.

I confirm that I have read this submission and believe that I have an appropriate level of expertise to confirm that it is of an acceptable scientific standard, however I have significant reservations, as outlined above.
